# The Importance of Alliance between Hematologists and Dentists: A Retrospective Study on the Development of Bisphosphonates Osteonecrosis of the Jaws (Bronj) in Multiple Myeloma Patients

**DOI:** 10.3390/dj9020011

**Published:** 2021-01-20

**Authors:** Christian Bacci, Alessia Cerrato, Virginia Dotto, Renato Zambello, Gregorio Barilà, Albana Liço, Gianpietro Semenzato, Edoardo Stellini, Gastone Zanette

**Affiliations:** 1 Clinical Dentistry Department of Neurosciences Via Giustiniani 1, University of Padova, 35128 Padova, Italy; cerrato.alessia92@gmail.com (A.C.); virginia.dotto@yahoo.it (V.D.); edoardo.stellini@unipd.it (E.S.); gastone.zanette@unipd.it (G.Z.); 2 Department of Medicine, Hematology and Clinical Immunology Section, University of Padova, 35128 Padova, Italy; renato.zambello@aopd.veneto.it (R.Z.); gregorio.barila@aopd.veneto.it (G.B.); albana.lico@unipd.it (A.L.); g.semenzato@unipd.it (G.S.)

**Keywords:** MRONJ, BRONJ, ONJ, osteonecrosis, myeloma, bisphosphonates

## Abstract

(1) Background: Multiple myeloma is a rare cancer that primarily affects the bone marrow. Osteoclasts are responsible for increased bone resorption and, therefore, bone destruction. Bisphosphonates are a class of drugs that can slow down bone resorption by reducing the number and action of osteoclasts. Intravenous injections of bisphosphonates (generally Zoledronic Acid) are administered to patients affected by Multiple Myeloma, but BRONJ is described as a serious side effect. This 5-year retrospective study aims to evaluate the efficacy of appropriate dental treatment protocols prior to initiating bisphosphonate therapy to prevent the development of BRONJ. (2) Methods: A total of 99 patients with symptomatic multiple myeloma were involved in this study (41–90 years, mean age 65 years, standard deviation 5 years). The data relating to the visits were tracked using a specific server and consulting the clinical reports. The AAOMS (American Association of Oral and Maxillofacial Surgeons) position was applied for both diagnosis and treatment. A total of 79 patients were examined before the administration of bisphosphonates (group A) and 20 after (group B). (3) Results: The entire sample required dental treatment: 23.2% underwent restorative therapy, 8% endodontic treatments, 44.4% tooth extractions. Periodontal disease was present in 41.4% of the patients. No osteonecrosis was observed in the first group, whereas BRONJ was found in five patients of the second one (25%) and two patients (10%) showed osteosclerotic areas under investigation [OR 0.026 (CI 0.0027 to 0.2454)]. (4) Conclusions: In the literature, there are no precise data about the prevalence of BRONJ. Despite the limitation of the present study, we point out that dental treatment before the treatment with intravenous bisphosphonates can help in reducing the incidence of BRONJ and good dental status is necessary for BRONJ prevention.

## 1. Introduction

Multiple Myeloma (MM) is a rare and serious tumor, which affects plasma cells. It constitutes 1% of all tumors, and it is typical in old age, with a median age at diagnosis of 65 [1,2,3].

Among hematological malignancies, however, it is relatively common (10–13% of hematological cancers) [1]. In the United States, the lifetime risk of getting MM is about 0.7% (American Cancer Society), with a greater risk for males than females, according to the data of the Italian Association for Cancer Research (AIRC) [3,4].

The incidence is 4.6–7.1 per 100,000 in the U.S. and 5.7 per 100,000 in Europe [5,6].

Multiple myeloma is a pathology of plasma cells that leads to the production of monoclonal immunoglobulins and the invasion and destruction of nearby bone tissue. Plasma cells, localized for the major part in the bone marrow, are the final result of B-Lymphocyte maturation, and are involved in the immune response of the human body. They are very important for the immune system, because the B cells change into plasma cells in case of infection, and these cells produce antibodies (also known as immunoglobulines) to defeat germs.

Diagnosis of Multiple Myeloma requires the presence of at least 10% of malignant plasma cells in the bone marrow; these neoplastic cells produce a monoclonal antibody, known as M-protein (monoclonal immunoglobulin protein), as well as other substances, which stimulate osteoclasts and lead to bone tissue resorption [4,7,8].

The M-protein could be IgG (55% of myeloma patients) and IgA (about 20%). Among those patients, 40% also have Bence Jones proteinuria. IgD myeloma amounts to 1% of cases. Sometimes, patients have no M-protein in blood and urine.

Multiple myeloma can be symptomatic or not: in the last case the condition is known as “Smoldering Myeloma” (SMM), and no chemotherapy or bisphosphonate treatment is required. SMM could be considered the connection between monoclonal gammopathy and active multiple myeloma.

MM can become symptomatic due to the development of Myeloma-related tissue impairment according to CRAB criteria that include: hyperCalcemia, Renal Insufficiency, Anemia, and Bone lytic lesions; the patient can also experience, with less frequency, recurrent infections, peripheral neuropathy and hyperviscosity syndrome.

According to the 2014 IMWG consensus, specific Myeloma treatment can be started in symptomatic patients but also in asymptomatic patients with high-risk disease (with 80% risk of progression in the next 2 years) defined by the presence of at least 60% of bone marrow malignant plasma cells or FLC ratio >100 or by the presence >1 focal lesion at MRI.

As far as the treatment is concerned, it consists of a combination of chemotherapy, radiation, stem cell transplant, biologic therapy and bisphosphonates therapy [9].

Osteoclasts are cells responsible for increased bone resorption and, therefore, bone destruction. Bisphosphonates are a class of drugs that can slow down bone resorption by reducing the number and action of osteoclasts. They also play a role in the prevention of some complications in MM, including hypercalcemia, pathologic fractures, and spinal cord compression. Bisphosphonates administration reduces the progression of cancer, decreases skeletal-related morbidity and, probably, pain [10].

More than 30 years have passed since bisphosphonates were placed on the mark and they have been used for many skeletal diseases. From a chemical point of view, these medicines are similar to pyrophosphate (PPi) and they have the characteristic of binding to the hydroxyapatite crystals of the bone (so bisphosphonate presence into bone depends on the percentage of hydroxyapatite binding sites). These drugs are primarily taken up where bone turnover is greater.

The core structure of BPs is analogue to PPi with a central nonhydrolyzable carbon. Distinct from PPi, BPs have a hydroxyl group linked to the carbon (R^1^). The flanking phosphate groups are responsible for hydroxyapatite affinity (these groups are also found in PPi), whereas the hydroxyl group increases the drug’s power to bind calcium.

The so-called R^2^ group bound to the central carbon is the most determining factor in the potency of BPs in inhibiting bone resorption. The presence of a nitrogen or amino group (like in Zoledronate and Pamidronate) increases the bisphosphonate’s antiresorptive potency relative to early non-nitrogen-containing bisphosphonates.

There are several RCTs that confirm the benefit of BFs therapy, in terms of survival. [11].

Among bisphoshonates, zoledronic acid appears to be better than placebo and etidronate as far as efficacy is concerned [10]. Pamidronic acid and Zoledroic acid are the most frequently used drugs for the treatment of myeloma-related skeletal disorders [10,12].

The possible side effects of bisphosphonates could be gastrointestinal symptoms, hypocalcemia, renal disfunction and osteonecrosis of the jaw [12].

BRONJ (Bisphosphonate-Related Osteonecrosis of the Jaws) can be defined as “A drug-related adverse reaction, characterized by progressive bone destruction and necrosis of both the mandible and the maxilla, in patients taking aminobisphosphonates or other drugs (such as antiresorptive and antiangiogenic), with no other predisposing conditions” (Italian Association of Maxillofacial Surgery, SICMF, and Italian Association of Oral Medicine and Pathology, SIPMO, 2014).

## 2. Materials and Methods

This 5-year retrospective study aims to evaluate the efficacy of appropriate dental treatment prior to initiating bisphosphonate therapy to prevent the development of BRONJ in a sample of patients with multiple myeloma. Only patients affected by symptomatic multiple myeloma require BFs treatment, to reduce skeletal-related events, such as pain and fractures [10]. This is the reason why patients affected by smoldering multiple myeloma could not be involved in this protocol.

Symptomatic multiple myeloma should be treated with bisphosphonate therapy, consisting of Zoledronate intravenous injections at a dosage calculated to renal function.

Ninety-nine patients with a diagnosis of symptomatic MM were enrolled in this study. From July 2011 to September 2015 the 99 patients, aged between 41 and 90 (mean age 65 years, standard deviation 5 years, 55 male and 44 female), underwent specific Myeloma treatment at the Department of Hematology and Clinical Immunology, Azienda Ospedaliera of Padua, and were visited by a dentist at the University of Padua Hospital.

All data relating to dental visits and treatments were tracked using a specific provider (Galileo) and by consulting the clinical reports. A table was created using Excel, were collected for every patient and the treatment required was noted, including also the oral hygiene status and the denture relining requirement, when reported by the clinician.

The guidelines provided by the American Association of Oral and Maxillofacial Surgeons (AAOMS) were applied for both diagnosis and treatment.

Among the entire sample size, 79 patients were visited before the first Zoledronate injection (group A) and 20 after starting the therapy. These 20 patients were visited because of oral problems developed during bisphosphonate treatment (group B).

## 3. Results

**Figure 1:**
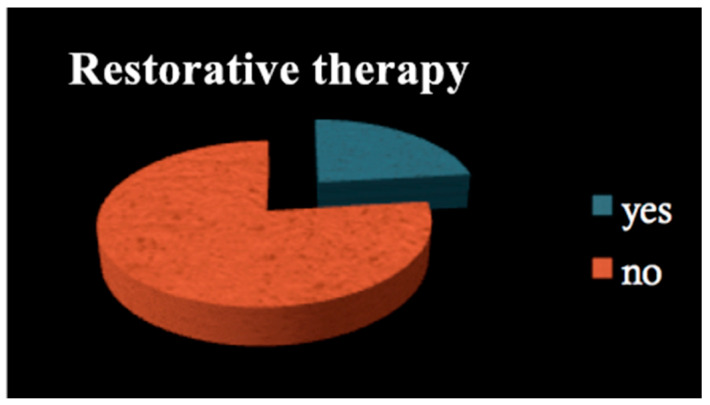
Restorative therapy.

**Figure 2:**
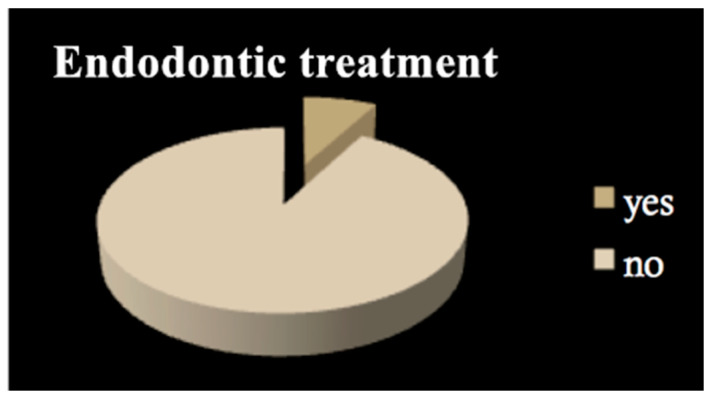
Endodontic treatment.

**Figure 3:**
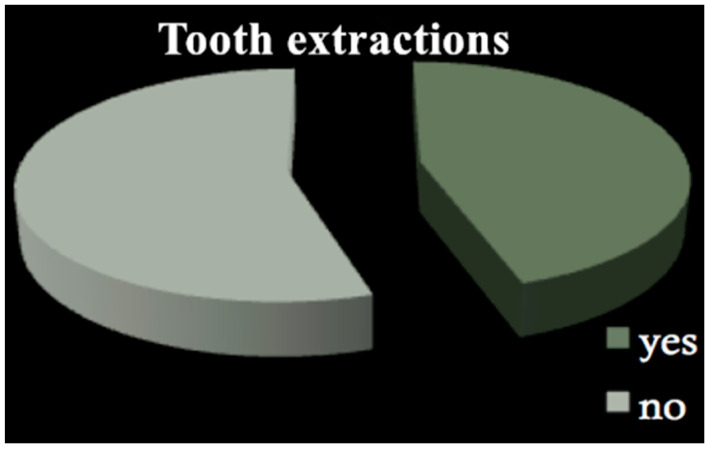
Tooth extractions.

**Figure 4:**
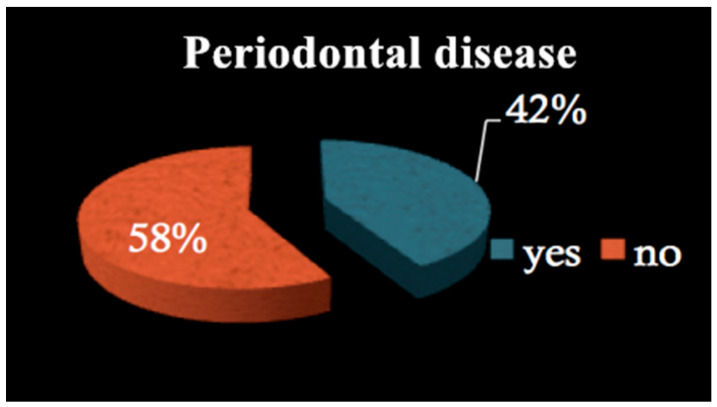
Periodontal disease.

All patients among both groups required dental treatment: 23.2% needed restorative therapy (Figure 1), 8% endodontic therapy (Figure 2) and 44.4% underwent tooth extractions (among these patients, 61.3% required multiple extraction, both in the mandible and in the maxilla, mean age 70 years old, sd = 10) (Figure 3). Periodontal disease affected 41.4% of the patients (Figure 4). Six patients of 99 were edentulous patients. Seven patients, all belonging to group A, required denture relining.

Oral hygiene status was described for 32 patients, 22 of group A and 10 of group B. Among the first group, only six patients had good oral hygiene (27.3%), whereas six patients of group B had an adequate oral hygiene level (60%).

No cases of ONJ were found in group A, but in group B there were five patients (25%) with ONJ, while in two patients (10%), osteosclerotic areas were found [OR 0.026 (CI 0.0027 to 0.2454)]. ONJ was observed in group B after tooth extraction, even if all the correct procedures were followed (full-thickness flap elevation, atraumatic surgery, antibiotic prophylaxis with amoxicillin and metronidazole, chlorhexidine rinses).

## 4. Discussion

BRONJ (Bisphosphonates Related Osteonecrosis of the Jaws) was firstly described in 2003, and the association between these conditions and bisphosphonate intake is reported by several authors. More questionable is the pathogenesis of BRONJ, which is still a matter of discussion [13].

A lot of hypotheses have been formulated: the inhibition of bone remodeling produced by BPs, the antiangiogenic properties of these drugs and the role of microorganisms are the most probable risk factors [12,14].

To date, no data on the exact prevalence of BRONJ are available. Some studies report a prevalence of between 1% and 12% in patients receiving intravenous bisphosphonates, while only a few studies rely on a large sample [15,16]. Some authors report an 8.9% prevalence of BRONJ, in patients with multiple myeloma or other malignant tumors, and current data confirm the results of Bamias et al., reporting a prevalence of 6.7% [17,18].

According to some other authors, the prevalence of BRONJ is on the contrary higher (Boonyapakorn reported a rate of 28%), and one of the mentioned reasons is the fact that the sample size is made for the 46% of patients affected by multiple myeloma, with highly-dosed cytostatics and corticosteroids [17,19].

Otherwise, the definition of ONJ is complex and it has been changed and reviewed lots of times during the past 10 years (also the name of this condition has changed from ONJ to BRONJ, to MRONJ). The first definition was formulated in 2007 by the AAOMS, and the diagnosis of BRONJ was closely related to the presence of exposed necrotic bone for more than 8 weeks in the oral cavity (Advisory Task Force on Bisphosphonate-Related Ostenonecrosis of the Jaws 2007) but at this time many things have changed and the presence of exposed necrotic bone is no more necessary for the diagnosis of BRONJ. The clinicians are aware of the etiology, the imaging and the stages of BRONJ, but some aspects, such as pathogenesis, role of fungal infections, and treatment, must be clarified.

According to the literature data, the median risk of BRONJ development, after bisphosphonates intravenous administration, in cancer patients ranges between 1 and 10% in the 2 years after the first injection [18,20,21].

This risk seems to be higher in patients treated with Zoledronate instead of Pamidronate, whereas no data are available concerning Ibandronate use [22,23].

The risk of developing BRONJ is higher in patients affected by multiple myeloma than in patients undergoing bisphosphonate therapy due to other diseases, for example, bone metastases of an advanced breast or prostate cancer. [23,24] Risk is extremely rare in patients taking bisphosphonate for metastases of other kinds of cancers (for example lungs, kidneys and pancreas) [20]. BRONJ can occur also in patients treated with low doses of oral BPs (for example for osteoporosis), but the risk is not so high.

Since 2007 there have been some guidelines for the management of patients affected by BRONJ, medical and surgical approaches have been compared, but the most useful treatment of this condition is undoubtedly its prevention.

This study was intended to evaluate the efficacy of correct dental treatment before starting bisphosphonate therapy, to prevent BRONJ development in a sample of multiple myeloma patients [25,26], but it is reasonable to consider that prevention should start before Zoledronate. In particular, performing a dental visit before the treatment with Zoledronic Acid, is the best way to avoid major complications in the future.

As reported in our results, no cases of ONJ were found in group A, in group B, five patients (25%) with ONJ were found, while in two patients (10%), osteosclerotic areas were found. It means that among patients visited during bisphosphonate therapy, 20% developed BRONJ, and 10% showed bone lesions. The risk of developing BRONJ was from 4 to 6 times lower in patients visited before starting the treatment with Zoledronate, compared to patients who did not receive a dental check-up before starting.

Our results showed that 44.4% of patients required dental extractions, mainly due to the presence of periodontitis, which affected 41.4% of all patients enrolled in this study. This subset was very similar to the prevalence of periodontitis among the common population, according to some epidemiological studies [27]. Considering that periodontitis is so widespread, it seems evident that a preventive dental visit should be mandatory before BF treatment, as several studies confirm that periodontal patients show a higher risk of BRONJ development [28].

Antibiotics were administered to all patients of group B before dental extractions because they were already taking bisphosphonates. No antibiotics were administered to patients of group A.

During the follow-up visits, furthermore, we noticed that the oral hygiene status was very improved in both groups and it is well known that a good dental status is essential before, during and after bisphosphonate administration [29]. The preventive dental visit could have maybe motivated these patients to correct oral hygiene. The importance of a good oral hygiene status is underlined by several studies, as a fundamental practice to prevent BRONJ development, like the necessity to undergo adequate dental care, at least twice a year if not more frequently [30,31]. That is an example of advice we gave to patients during our visits, and it could have contributed to obtaining such satisfying results in BRONJ prevention.

As far as dental maintenance care is concerned, it is important to consider that the pattern of myeloma can be varied such as the oral presentation, which could be one of the signs of disease progression [32,33], so a dental visit should be mandatory, both before and after BF therapy.

## 5. Conclusions

A limit of this study could be that we did not take into consideration the contemporaneous intake of other drugs, such as corticosteroids, or other diseases, like diabetes, that could influence.

It is fundamental the coordination between hematology and dentistry to perform adequate dental treatment and planning.

According to several studies [30], BRONJ seems to be more frequent after long-term use of BFs. Another limit of this study is that the duration of the treatment was not taken into consideration.

Within the limits of this study, we report that dental diagnosis and adequate treatment prior to initiating treatment with intravenous bisphosphonates can help reduce the incidence of BRONJ.

Obviously, to reach scientific evidence as always, RCTs and long-term follow-up are needed to confirm or refute these results.
